# Review of the cost-effectiveness of surveillance for hereditary pancreatic cancer

**DOI:** 10.1007/s10689-024-00392-1

**Published:** 2024-05-25

**Authors:** Louise Wang, Rachel Levinson, Catherine Mezzacappa, Bryson W. Katona

**Affiliations:** 1 Section of Digestive Diseases, Yale School of Medicine, New Haven, CT USA; 2 VA Connecticut Healthcare System, West Haven, CT USA; 3 Division of Gastroenterology and Hepatology, University of Pennsylvania Perelman School of Medicine, 3400 Civic Center Blvd. 751 South Pavilion, Philadelphia, PA 19104 USA

**Keywords:** Hereditary pancreatic cancer, Pancreatic surveillance, Germline genetic risk, MRI, Endoscopic ultrasound, New-onset diabetes, Cost-effectiveness

## Abstract

Individuals with hereditary pancreatic cancer risk include high risk individuals (HRIs) with germline genetic susceptibility to pancreatic cancer (PC) and/or a strong family history of PC. Previously, studies have shown that PC surveillance in HRIs can downstage PC diagnosis and extend survival leading to pancreatic surveillance being recommended for certain HRIs. However, the optimal surveillance strategy remains uncertain, including which modalities should be used for surveillance, how frequently should surveillance be performed, and which sub-groups of HRIs should undergo surveillance. Additionally, in the ideal world PC surveillance should also be cost-effective. Cost-effectiveness analysis is a valuable tool that can consider the costs, potential health benefits, and risks among various PC surveillance strategies. In this review, we summarize the cost-effectiveness of various PC surveillance strategies for HRIs for hereditary pancreatic cancer and provide potential avenues for future work in this field. Additionally, we include cost-effectiveness studies among individuals with new-onset diabetes (NoD), a high-risk group for sporadic PC, as a comparison.

**Graphical Abstract:**

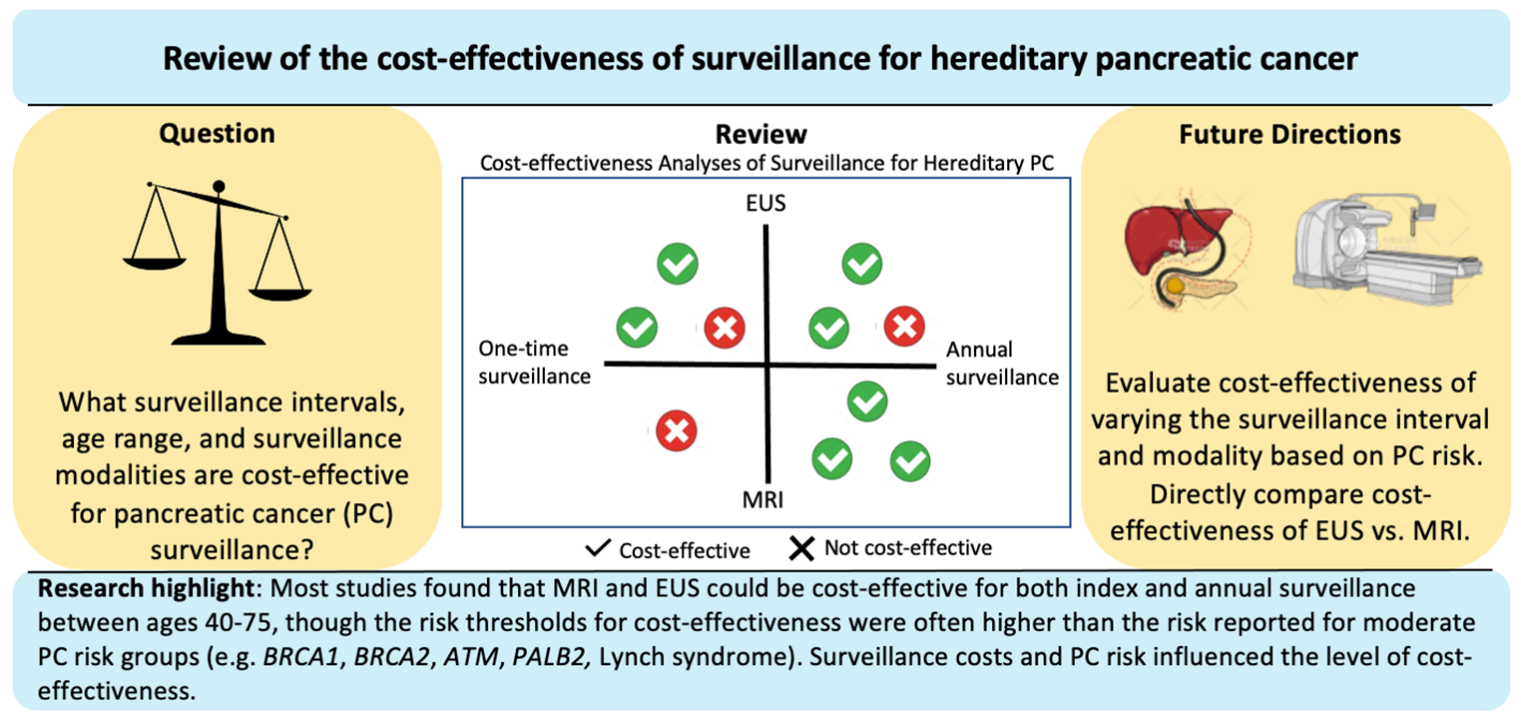

**Supplementary Information:**

The online version contains supplementary material available at 10.1007/s10689-024-00392-1.

## Introduction

Pancreatic cancer (PC) is the third leading cause of cancer death in the United States [1]. The majority of PC is metastatic at the time of diagnosis with poor survival (5-year survival rate: 3.2%) [2]. In contrast, PC diagnosed at local stages can often be treated surgically, resulting in improved survival (5-year survival rate: 44.3%) [2]. In fact, the most localized stage IA PC has a 5-year survival of over 80% [3], demonstrating how it is crucial to focus efforts on identifying PC at earlier stages to prevent PC-related deaths.

Approximately 10% of PC is hereditary due to either known germline genetic susceptibility and/or family history of PC. Genes associated with increased PC risk include hereditary breast cancer genes (*BRCA1, BRCA2, ATM*, and *PALB2*), *CDKN2A* (familial atypical multiple mole-melanoma syndrome - FAMMM), Lynch syndrome genes (*MLH1, MSH2/EPCAM*, *MSH6*), *STK11* (Peutz-Jeghers syndrome), *TP53* (Li-Fraumeni syndrome), and hereditary pancreatitis genes such as *PRSS1* [4]. Additionally there is increased risk amongst families with familial pancreatic cancer (FPC), in which there are at least two relatives with PC who are directly related to one another, with the proband being a first-degree relative of one of the affected individuals, without a known pathogenic germline variant (PGV) in a PC risk gene. FPC may confer a lifetime PC risk estimate of up to 20–40% depending on the number of affected first-degree relatives [4]. In contrast, 90% of PC is considered sporadic, arising in the absence of both a known genetic predisposition and a strong family history of PC. High-risk sporadic groups include individuals with new-onset diabetes (NoD) diagnosed after age 50. The NoD in this cohort is thought to be a subclinical sign of existing PC and this risk is highest within the first three years after diabetes diagnosis, conferring an 8-fold higher risk than that of the general population [5, 6].

## Cost-effectiveness analyses

**Fig. 1:**
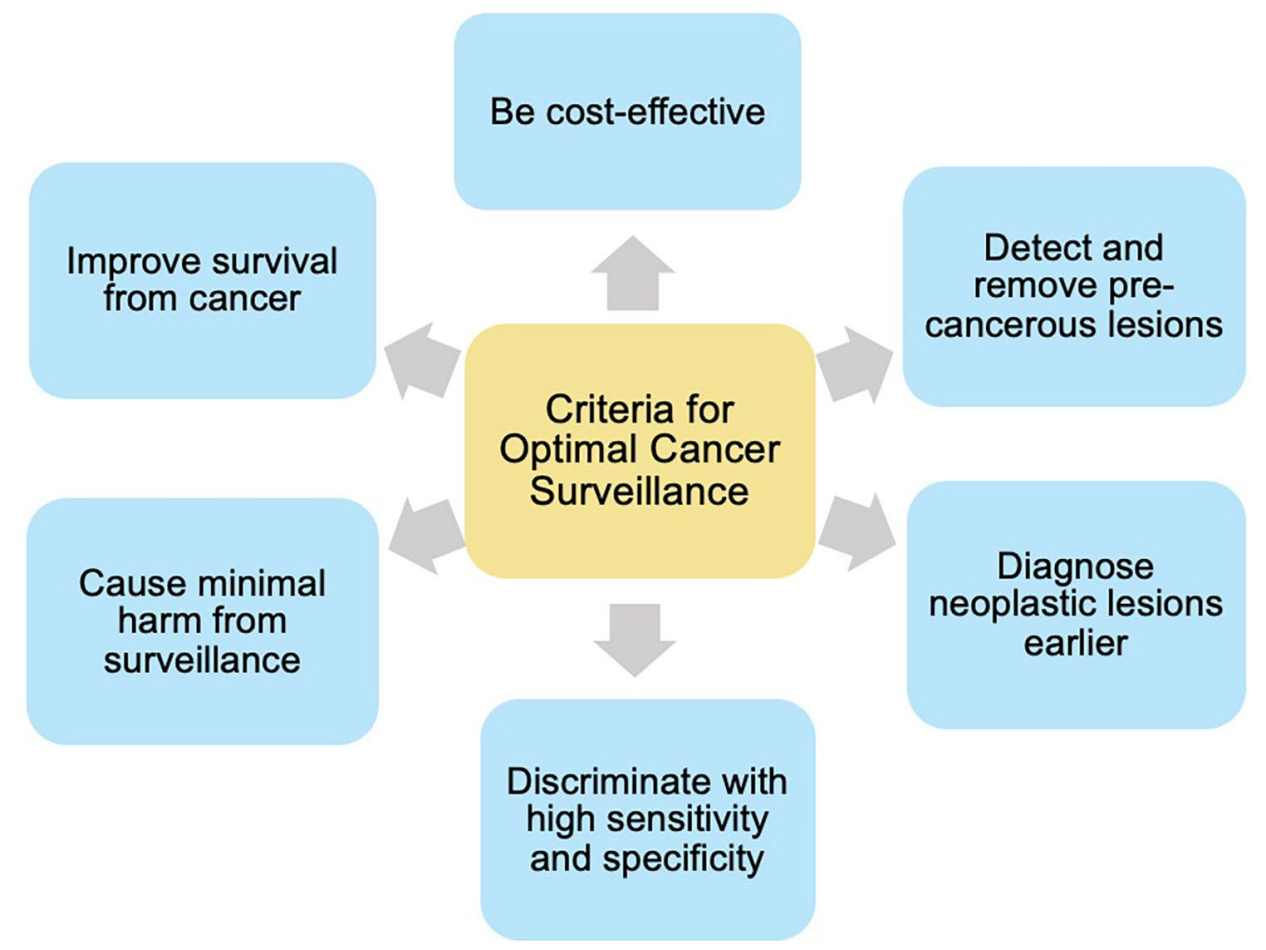
Criteria for optimal cancer surveillance

High-risk individuals (HRIs) with either a PGV or FPC are at increased risk of PC, and results from the American Cancer of the Pancreas Screening (CAPS) study showed that 7% had neoplastic progression (either PC or high-grade dysplasia) over a 16-year period of surveillance [7]. However, for HRIs there remains debate about the appropriate surveillance strategy to employ, with uncertainty about the optimal imaging modality, age to start and stop surveillance, and surveillance interval [8–11]. For PC surveillance tests to be considered optimal, they must satisfy the following criteria: (i) allow for earlier detection of neoplastic lesions or detection/removal of pre-cancerous lesions, (ii) improve survival from cancer, (iii) have high sensitivity and specificity, and (iv) cause minimal harm (Fig. 1). Additionally, an optimal PC surveillance test should also weigh the benefits, risks and costs of each strategy compared with the current status quo and ideally be cost-effective.

Cost-effectiveness analyses (CEAs) for cancer surveillance consider the trade-off between the potential health benefits and the costs of surveillance to another strategy, such as no surveillance. Health benefits of surveillance are typically measured in either (i) life-years gained, the added years of life from an intervention or (ii) quality-adjusted life years (QALYs), which integrate the quality of life over time [12]. We quantify the quality of life based on utility values, which assign a number between 0 (death) to 1 (perfect health), with values in between representing various health conditions [13]. For example, compared to a perfect health state, receiving a total pancreatectomy would lower the utility value due to factors such as operative risks, impaired physical functioning, and postoperative diabetes [14].

Most CEAs are measured from a healthcare perspective, which considers the monetary costs of healthcare, such as costs of PC surveillance and treatment. In contrast, a societal perspective incorporates non-healthcare costs such as the loss of job productivity [15, 16] or the cost of premature death [17]. Various decision strategies can be compared using an incremental cost-effectiveness ratio (ICER), which is a ratio of the difference in costs versus the difference in QALYs (or life-years) between surveillance (e.g. endoscopic ultrasound (EUS) or magnetic resonance imaging (MRI)) and no surveillance. An intervention is typically deemed cost-effective when the ICER is below the willingness-to-pay (WTP) threshold, which ranges from $50,000 to $150,000 per QALY gained using World Health Organization recommendations based on per capita country levels [18, 19].

### Current guidelines and modalities of PC surveillance

In HRIs, PC surveillance has been shown to downstage PC and substantially prolong survival after PC diagnosis. Specifically, in the multicenter Cancer of Pancreas Screening (CAPS)-5 study, the majority of PC diagnosed through the surveillance program from 2014 to 2021 was downstaged compared to those not undergoing active surveillance, with the majority of patients with surveillance detected PCs being stage I (78%) with resectable (89%) cancers [20]. Amongst the full CAPS1-5 cohorts, median survival was 9.8 years for surveillance detected PCs, whereas in contrast, 85% of PC detected outside surveillance were stage IV, with a median overall survival of 1.5 years [20]. Klatte et al. similarly found substantial improvements in resectability, earlier detection, and survival in a surveillance program in the Netherlands among a cohort of *CDKN2A* PGV carriers [21].

Currently, MRI and EUS are favored imaging modalities for HRIs undergoing PC surveillance, often as complementary, interchangeable approaches. MRI is more sensitive for evaluation of cystic lesions, and EUS is better for detection of solid lesions [8]. Computerized tomography (CT) scans are not recommended for surveillance in HRIs given their lower sensitivity for detection of pancreatic lesions [22]. In addition to imaging tests, consideration can also be made of screening HRIs for diabetes annually with either a hemoglobin A1c or fasting blood glucose given the association between subclinical PC and NoD [6, 11]. While there has been extensive research on other blood-based biomarkers for PC surveillance in HRIs, at this time no such tests are regularly utilized in clinical practice. In fact, data from the first commercially available blood test specifically designed for PC early detection showed unfavorable positive predictive value [23].

Despite the benefits of surveillance, guidelines for surveillance vary depending on the lifetime risk of PC. For individuals at the highest risk, such as those with FAMMM or Peutz-Jeghers syndrome, consensus guidelines [8–11, 24] vary some, but typically recommend starting surveillance between ages 30–40. For individuals at moderate risk, there is more variation in guidelines. For example, the ASGE recommends that appropriately-aged individuals with a *PALB2*, *BRCA1*, or *BRCA2* PGV undergo PC surveillance regardless of family history, while other guidelines recommend surveillance of these individuals only if they also have a family history of PC in either a first- or second-degree relative [9, 10, 25]. In addition to questions about risk thresholds for surveillance, many of the other inputs to determine cost-effectiveness, including surveillance interval, age to start surveillance, and costs, are not standardized.

### Literature review on cost-effectiveness of PC surveillance

We conducted a comprehensive literature search in PubMed to find relevant articles published from database inception to February 16, 2024. Search terms for HRIs included keywords related to pancreatic cancer, pancreatic screening, pancreatic surveillance, and cost-effectiveness analyses. Search terms for individuals with NoD included additional keywords related to diabetes. A full list of keywords and search strategy are provided in Supplementary Table S1. Additional articles were searched based on relevant cited and similar articles from the initial search query. Studies included in this review met the following criteria: (i) included a study population of individuals at high-risk for PC (risk associated with familial pancreatic cancer, genetic predisposition, or NoD) (ii) investigated imaging modalities for PC surveillance, and (iii) conducted a cost-effectiveness analysis evaluating both costs and benefits of surveillance.

### Cost-effectiveness of PC surveillance in individuals with familial or genetic risk

We identified eight cost-effectiveness studies of hereditary PC, published between 2002 and 2023 (Table 1). The majority were based on data from the United States, and the rest of the studies came from Japan, Denmark, and the Netherlands. Six out of the eight studies found that surveillance with either EUS or MRI was cost-effective compared to no surveillance at WTP of $50,000-$100,000/QALY [26–31]. Two of these studies [26, 32] did not specify their WTP threshold and surveillance strategies were either completely dominated (e.g. not cost-effective at any baseline input) or of minimal cost (e.g. <$50,000/QALY). Most studies [27–33] (*n* = 7) showed a third party payer perspective compared to total costs from a societal perspective, and four [27, 28, 30, 32] focused on an annual surveillance exam versus a one-time exam.

**Table 1:** Study characteristics for high-risk individuals with genetic predisposition or FPC undergoing pancreatic cancer surveillance

Study	Country	Population	Population source	Lifetime risk of PC	Follow-up	Surveillance strategies	ICER	WTP	Conclusion (base-case analysis)	Threshold analyses
Rulyak et al., 2002 [26]	US	Age 50, FPC kindred	Simulated cohort	Not specified	Lifetime	No surveillance One-time EUS	No surveillance: reference One-time EUS: *Direct costs*: $16,855/LYS^a^ *Total costs*: dominates^b^	Not specified	One-time EUS is cost-effective from a healthcare perspective and cost-saving from a societal perspective	Minimum prevalence of dysplasia needs to be ≥ 13% and sensitivity of EUS ≥ 75% for surveillance to be cost-effective
Rubenstein et al., 2007 [32]	US	Age 45, male, FPC kindred, with chronic pancreatitis on prior EUS	Simulated cohort	20%	Until age 90 or death	No surveillance Annual EUS Annual EUS/FNA PTP	No surveillance dominates	Not specified	No surveillance is the most cost-effective strategy	If specificity of EUS/FNA is 100% and mortality from localized cancer < 71%, annual EUS/FNA provides the most QALYs
Joergensen et al., 2016 [27]	Denmark	Age > 30, *PRSS1* mutation or HP kindred Age 50^c^, FPC kindred	Surveillance program	40%	2006–2014	No surveillance Annual EUS	No surveillance: reference Annual EUS: *HP cohort*: $58,647/QALY *FPC cohort*: $47,867/QALY	$50,000	Annual EUS is cost-effective for patients from FPC kindreds	N/A
Corral et al., 2019 [28]	US	Age 40, HRIs	Simulated cohort	≥ 5%	Lifetime	No surveillance Annual EUS Annual MRI	No surveillance: reference Annual EUS: $13,200/QALY Annual MRI: dominates	$100,000	Annual MRI is the most cost-effective strategy, but annual EUS is also cost-effective	If MRI >$1,600 or RR > 20, then annual EUS is the most cost-effective strategy
Kowada, 2020 [33]	Japan	Age 50, FPC kindred	Simulated cohort	32-fold increased risk	Lifetime	No surveillance One-time imaging with either: Abdominal US EUS MRI CT PET	No surveillance: dominated^d^ One-time abdominal US: reference One-time EUS: $101,026/QALY One-time MRI: $214,488/QALY One-time CT: dominated One-time PET: dominated	$50,000	One-time abdominal US is the most cost-effective strategy	If yearly incidence of PC is 0.8%-1.6%, one-time EUS is the most cost-effective strategy If yearly incidence of PC > 1.6%, one-time MRI is the most cost-effective strategy
Kumar et al., 2022 [29]	US	Age 55, HRIs	Simulated cohort	≥ 5%	Lifetime	No surveillance One-time EUS	No surveillance: reference One-time EUS: $82,669/QALY	$100,000	One-time surveillance with EUS is cost-effective at ≥ 9.6% lifetime PC risk	If lifetime PC risk ≥ 6.75% with normal life expectancy post-resection, one-time surveillance with EUS is cost-effective If lifetime PC risk is 5%, one-time surveillance with EUS is not cost-effective
Ibrahim et al., 2023 [30]	Netherlands	Age 45, *CDKN2A*-p16-*Leiden* mutation	Simulated cohort based on surveillance program data	37.6%	Lifetime	No surveillance Annual MRI	No surveillance: reference Annual MRI: €14,000/QALY	€50,000	Annual surveillance with MRI and optional EUS is cost-effective	Lifetime PC risk needs to be ≥ 10% for surveillance to be cost-effective
Peters et al., 2023 [31]	US	Age 40–70 in 5-year increments, HRIs^e^	Simulated cohort	> 2-fold increased risk^e^	Lifetime	No surveillance Annual MRI MRI every 2 years MRI every 5 years One-time MRI Stratified by sex	No surveillance: reference Annual MRI:^f^ *Male CDKN2A* $82,000/QALY (RR 12.33, age 55) *Male STK11* $69,000/QALY (RR 28, age 40) *Female STK11* $45,000/QALY (RR 28, age 45) MRI every 2 years: dominated MRI every 5 years: dominated One-time MRI: dominated	$100,000	For men, annual surveillance with MRI (+ EUS/FNA if abnormal) is cost-effective for *CDKN2A* and *STK11* cohorts starting at ages 55 and 40, respectively For women, annual surveillance with MRI (+ EUS/FNA if abnormal) is cost-effective for *STK11* cohorts starting at age 45	If WTP is $200,000, one-time surveillance with MRI is cost-effective for men with 5-10-fold increased lifetime PC risk If surveillance costs are halved, annual surveillance with MRI is cost-effective for women in the *CDKN2A* cohort

*Abbreviations*: ICER = incremental cost-effectiveness ratio; WTP = willingness to pay threshold; FPC = familial pancreatic cancer; EUS = endoscopic ultrasound; LYS = life-year saved; QALY = quality-adjusted life year; FNA = fine-needle aspiration; PTP = prophylactic pancreatectomy; HP = Hereditary pancreatitis; HRIs = high-risk individuals; PC = pancreatic cancer; RR = relative risk; MRI = magnetic resonance imaging; US = abdominal ultrasound; CT = computed tomography; PET = positron emission tomography

^a^Life years, rather than QALYs, were measured in this study

^b^Dominant strategy: the strategy is more effective and less costly than the compared strategy

^c^Age 50 or five years before age when youngest family member diagnosed with PC

^d^Dominated strategy: the strategy is more costly and less effective than the compared strategy

^e^ HRIs in this study were eight cohorts of patients with the following pathogenic germline variants (PGV): PALB2 (RR 2.33), BRCA1 (RR 2.58), Lynch syndrome PGVs (RR 3.55), ATM (RR 5.71), BRCA2 (RR 6.2), TP53 (RR 6.7), CDKN2A (RR 12.33), STK11 (RR 28)

^f^ ICERs reported for cost-effective strategies that were closest to WTP

Four studies [26, 27, 29, 32] compared EUS to no surveillance. Joergensen et al. [27] found that from 2006 to 2014, annual surveillance with EUS was cost-effective at a WTP of $50,000/QALY among a real-world cohort with FPC kindreds (baseline age > 50) but not in individuals with a *PRSS1* PGV or hereditary pancreatitis kindreds (baseline age > 30), noting that for the latter group, the analysis would influence their future prospective cohorts to begin at a baseline age of 40. These individuals also had higher rates of smoking than the general population, a known risk factor for PC. Additionally, two studies that evaluated a one-time surveillance exam with EUS (with possible endoscopic retrograde cholangiopancreatography (ERCP)) were cost-effective compared with no surveillance, but base inputs included high dysplasia rates [26], high lifetime PDAC probabilities [29], or long survival post-pancreatic resection. In contrast, Rubenstein et al. [32] concluded that annual surveillance with EUS or EUS with fine-needle aspiration (FNA) was not cost-effective in individuals with chronic pancreatitis and first-degree relatives with PC (20% lifetime PC risk). Their findings were largely because the annual mortality rates were similar between PC cases discovered at local and distant stages, and only at an EUS specificity of 100% and mortality from local cancer less than 71% would EUS/FNA be cost-effective [32]. However, given the study was published in 2007 and the increased survival and improved stage shifts discovered under surveillance programs recently [7], it would be reasonable to repeat this analysis with current resection and survival rates.

Two studies [30, 31] evaluated MRI surveillance with optional EUS compared to no surveillance. Peters et al. [31] evaluated surveillance strategies of MRI (one time, every 5 years, every 2 years, and annually) with follow-up EUS for eight separate high risk groups (*BRCA1* and *BRCA2, PALB2, ATM*, Lynch syndrome, *TP53*, *CDKN2A*, and *STK11*) and varied their starting ages (40–70 years old). Overall, one-time MRI or MRI every 2 or 5 years was dominated. For men, at a WTP of $100,000/QALY, annual MRI surveillance was cost-effective for the high-risk cohorts (RR 12, *CDKN2A*) starting at age 55 and highest-risk cohorts (RR 28, *STK11*) starting at age 40. In contrast for women, annual MRI surveillance was cost-effective for only the highest-risk cohort (RR 28, *STK11)* starting at age 45. No surveillance strategies were cost-effective for moderate risk and low risk individuals (*PALB2*, *BRCA1*, *BRCA2*, Lynch syndrome, and *TP53*) and ICERs ranged from $330,000 – $48.5 million/QALY. Among *CDKN2A* carriers, Ibrahim et al. [30] found that MRI surveillance (with optional EUS) was cost-effective compared to no surveillance at a WTP of €50,000/QALY among patients with a lifetime risk of PC ≥ 10%.

Two studies compared EUS versus MRI versus no surveillance. Corral et al. [28] found that annual MRI would be more cost-effective at a WTP of $100,000/QALY when the relative risk of PC is between 5 and 20 fold higher than the general population (annual incidence of 0.04% in the general US population), while EUS would be more cost-effective at a relative risk of more than 20 or if MRI costs exceeded $1,600. Kowada et al. [33] evaluated a one-time surveillance comparing abdominal ultrasound, EUS, CT, MRI, or positron emission tomography (PET) surveillance for a Japanese population and found that at the base-case scenario, abdominal ultrasound would be the most cost-effective approach at a WTP of $50,000/QALY. However, higher annual PC incidence would alter the choice of surveillance modality, with moderate incidence (0.8–1.6%) favoring EUS and high incidence (> 1.6%) favoring MRI. These two studies reached different conclusions favoring EUS or MRI, in part because the costs of surveillance strategies differed between the two countries. In the US study, EUS was more expensive, making it more cost-effective only for the highest-risk groups. In contrast, MRI was more costly in the Japanese study and cost-effective only in populations with the highest PC incidence.

### Cost-effectiveness of PC surveillance in individuals with new-onset diabetes

As a comparator we also examined cost-effectiveness analyses of PC surveillance among individuals with NoD, who are also at increased PC risk [34, 35] (Table 2). Both studies assumed a stage shift to detection of 30–40% of cancers in the local, resectable stages after surveillance, similar to results reported in prospective cohorts of genetically HRIs who had undergone surveillance [7]. Schwartz et al. [34] found that a one-time CT scan was cost-effective at a WTP of $100,000/QALY with a 0.82% probability of PDAC within the first three years of diabetes diagnosis. Wang et al. [35] varied risk thresholds of the individuals with NoD and found that a one-time screen with MRI (and follow-up EUS/FNA if a positive screen) was cost-effective at 1% (WTP $150,000/QALY) and 2% risk thresholds (WTP $100,000/QALY). Of note, the reported risk necessary for a one-time surveillance exam to be cost-effective among the NoD cohort was lower compared to the risk threshold necessary for surveillance to be cost-effective among genetic or familial HRIs, likely reflecting the concentrated risk of PC within the first three years of a diabetes diagnosis rather than a lifetime risk among individuals in the latter group.

**Table 2:** Study characteristics for high-risk individuals with new-onset diabetes undergoing pancreatic cancer surveillance

Study	Country	Population	Population source	3-year risk of PC	Follow-up	Surveillance strategy	ICER	WTP	Conclusion (base-case analysis)	Threshold analyses
Schwartz et al., 2021 [33]	US	Age ≥ 50, HRIs^a^ with NoD	Simulated cohort	≥ 0.5%	Lifetime	No surveillance One-time CT	No surveillance: reference One-time CT: $65,076/QALY	$100,000	One-time CT surveillance is cost-effective in HRIs with NoD	Minimum threshold of > 25% screen-detected PC cases need to be resectable for one-time CT scan to be cost-effective
Wang et al., 2022 [34]	US, UK	Age 66, HRIs^b^ with NoD	Simulated cohort	0.5%-5.0%	Lifetime	No surveillance One-time MRI	No surveillance: reference One-time MRI: Between $5,407/QALY (5% risk) to $290,132/QALY (0.5% risk)	$100,000 to $150,000	One-time surveillance with MRI (+ EUS/FNA if abnormal) for HRIs with NoD is cost-effective at ≥ 1% 3-year risk if WTP is $150,000 and at ≥ 2% 3-year risk if WTP is $100,000	If > 47.6% of PC cases are local at time of diagnosis, one-time MRI for > 0.5% 3-year risk is cost-effective

*Abbreviations*: ICER = incremental cost-effectiveness ratio; WTP = willingness to pay threshold; HRIs = high-risk individuals; NoD = new-onset diabetes; QALY = quality-adjusted life year; CT = computerized tomography; PC = pancreatic cancer; MRI = magnetic resonance imaging; EUS = endoscopic ultrasound; FNA = fine-needle aspiration

^a^Enriching New-Onset Diabetes for Pancreatic Cancer (END-PAC) score > 0

^b^Based on The Health Improvement Network (THIN) and END-PAC scores

### Areas of uncertainty and future directions

While most studies found that PC surveillance could be cost-effective, the risk thresholds identified for the surveillance to be cost-effective were often higher (e.g. ≥16% prevalence of dysplasia on an EUS in the surveillance population or > 10% lifetime risk of PC) than some of the reported lifetime risks for various moderate PC risk PGVs (e.g. *BRCA1*, *BRCA2*, *ATM*, *PALB2*, Lynch syndrome). This suggests that surveillance would only be appropriate and cost-effective for select high-risk groups and not all PGV/familial groups at risk for hereditary PC. Additionally, one study [31] firmly concluded that surveillance was not cost-effective in moderate risk groups. Given the heterogeneity of risk among HRIs, cost–effectiveness analyses offer a way to personalize surveillance: at the highest risk such as individuals with FAMMM (*CDKN2A*) or Peutz-Jeghers syndrome (*STK11*), it would be conceivable to conduct surveillance more frequently using more costly methods, while individuals at lower risk could potentially have longer surveillance intervals or utilize less costly, but also less sensitive surveillance methods, depending on their level of PC risk. For individuals with NoD, it is conceivable for a one-time surveillance for PC to be sufficient given the concentrated risk within 3 years of the diabetes diagnosis.

Furthermore, the optimal modality and frequency of surveillance remains to be determined. In the future, there need to be more direct comparisons between EUS and MRI to nuance surveillance decisions more appropriately given the decreasing costs and increasing performance of imaging. Of note, the studies did not uniformly explore the consequences of increased surveillance that could result in increased false positives and the potential resulting downstream procedures such as pancreatectomy or a Whipple procedure, which have their own disutility and associated mortality. Similarly, surveillance could also trigger the detection and subsequent removal of precancerous lesions such as cysts with high grade dysplasia, decreasing risk of PC rather than stage shifting to earlier cancer stages.

To build the most current cost-effectiveness analyses, we also need to update the models with ongoing surveillance data from prospective surveillance cohorts such as CAPS-5 [20] and European cohorts [21], to provide real-world data regarding the incident cancers detected, stage shift, and survival. We also need to stratify these results by HRI syndrome given the heterogeneity of PC risk.

To our knowledge, this is the first review of cost-effectiveness studies on hereditary pancreatic cancer. Given the scarcity of studies, we chose to report all published manuscripts on this topic. Nevertheless, we acknowledge that there was heterogeneity in the inputs such as differences in WTP thresholds, surveillance techniques, costs, probabilities, and utilities among the studies. As more of these cost-effectiveness analyses are published, future reviews can choose to be more discriminatory based on the quality and standardization of the inputs.

Historically, countries such as the United Kingdom and Germany have routinely used cost thresholds to help guide coverage of services and reimbursements. However, cost-effectiveness analyses are not routinely part of the decision-making process for medical goods and services in the United States, in part because of the negative societal views about the distribution of resources, multi-payer system with many stakeholders, and abstract nature of these analyses. However, given the rising costs of healthcare, it may be reasonable to consider using cost-effectiveness analyses to best focus the implementation of our surveillance strategies on the greatest good for the greatest number of individuals [36].

## Conclusion

In this literature review, we highlight existing published manuscripts that evaluate the cost-effectiveness of PC surveillance among genetic or familial HRIs and compared these studies with the existing literature on CEAs for individuals with NoD. Most of the studies that evaluated surveillance in HRIs found either EUS, MRI, or both had the potential to be cost-effective compared to no surveillance. However, some of the risk thresholds chosen suggest surveillance might only be cost-effective in select PGV/familial groups with high lifetime risk (e.g. >10% or RR > 12). The most important inputs included a high lifetime risk of PC or pancreatic dysplasia, high life expectancy post-pancreatic resection, or performance characteristics of the surveillance test. When directly comparing EUS and MRI and other surveillance modalities, no surveillance strategy was dominant and the imaging strategy of choice depended on the risk of PC and cost of the surveillance strategy, which varied globally. Future cost-effectiveness studies are needed to compare the cost-effectiveness of EUS and MRI as surveillance strategies in prospective cohorts with stratified PC risk, incorporating dynamic changes to the costs and test performance over time.

### Electronic supplementary material

Below is the link to the electronic supplementary material.

Supplementary Material 1

## Data Availability

No datasets were generated or analysed during the current study.
