# Supplementary material for: Review of the cost-effectiveness of surveillance for hereditary pancreatic cancer
Source: Fam Cancer. 2024 May 25;23(3):351–60. doi: 10.1007/s10689-024-00392-1 (PMC11255025; doi:10.1007/s10689-024-00392-1)
Supplement: Supplementary file 1 — Supplementary Material 1 [file 10689_2024_392_MOESM1_ESM.docx]

**Supplementary Table S1**. Search strategy and results.

**Keywords – Genetically High-risk Individuals**

**Keywords – New-onset Diabetes**

|  | P | I | O |
| --- | --- | --- | --- |
|  | Patients at High Risk for Pancreatic Cancer | Pancreatic Screening | Cost effectiveness |
| Keywords | “Pancreatic Neoplasm”  “Pancreas Neoplasm”  “Cancer Pancreas”  “Pancreas Cancers”  “Pancreas Cancer”  “Pancreatic Cancer”  “Pancreatic Adenocarcinoma”  “Familial Pancreatic Cancer”  “Familial Pancreatic Adenocarcinoma” | “Pancreatic Cancer Surveillance”  “Pancreas Surveillance”  “Pancreatic Cancer Screening”  “Cancer Early Detection”  “Cancer Screening”  “Cancer Screening Test”  “Early Diagnosis of Cancer”  “Cancer Early Diagnosis” | “economic analysis”  “economic evaluation”  “economic model”  “cost analysis” |
| MeSH | "Pancreatic Neoplasms"[Mesh] | "Early Detection of Cancer"[Mesh] | "Cost-Effectiveness Analysis"[Mesh] |

|  | P | I | O |
| --- | --- | --- | --- |
|  | Patients with New Onset Diabetes | Pancreatic Cancer Screening | Cost effectiveness |
| Keywords | “Type 2 Diabetes Mellitus”  “Type 2 Diabetes”  “Adult-Onset Diabetes Mellitus”  “New-Onset Diabetes” | “Pancreatic Cancer Surveillance”  “Pancreatic Cancer Screening”  “Cancer Early Detection”  “Cancer Screening”  “Cancer Screening Test”  “Early Diagnosis of Cancer”  “Cancer Early Diagnosis”  “Early Detection” | “Economic analysis”  “Economic evaluation”  “Economic model”  “Cost analysis” |
| MeSH | "Diabetes Mellitus"[Mesh] | "Early Detection of Cancer"[Mesh] | "Cost-Effectiveness Analysis"[Mesh] |

**Search Strategy – Genetically High-risk Individuals (23 results)**

("Pancreatic Neoplasms"[Mesh] OR "Pancreatic Neoplasms/diagnosis"[Mesh] OR "Pancreatic Neoplasms/diagnostic imaging"[Mesh] OR "Pancreatic Neoplasms/prevention and control"[Mesh] OR “Pancreatic Neoplasm” OR “Pancreas Neoplasm” OR “Pancreatic Cancer” OR “Pancreatic Adenocarcinoma” OR “Familial Pancreatic Cancer” OR “Familial Pancreatic Adenocarcinoma”) AND ("Early Detection of Cancer"[Mesh] OR “Pancreatic Cancer Surveillance” OR “Pancreatic Cancer Screening” OR “Pancreatic Screening” OR “Pancreas Surveillance” OR “Cancer Early Detection” OR “Cancer Screening” OR “Cancer Screening Test” OR “Early Diagnosis of Cancer” OR “Cancer Early Diagnosis” OR “Early Detection”) AND ("Cost-Effectiveness Analysis"[Mesh] OR “cost-effectiveness” OR “cost effectiveness” OR “economic analysis” OR “economic evaluation” OR “economic model” OR “cost analysis” OR “cost effectiveness” OR “cost-effectiveness analysis”)

**Search Strategy – New-onset Diabetes (5 results)**

("Diabetes Mellitus"[Mesh] OR “Type 2 Diabetes Mellitus” OR “Type 2 Diabetes” OR “Adult-Onset Diabetes Mellitus” OR “New-Onset Diabetes”) AND ("Pancreatic Neoplasms"[Mesh] OR "Pancreatic Neoplasms/diagnosis"[Mesh] OR "Pancreatic Neoplasms/diagnostic imaging"[Mesh] OR "Pancreatic Neoplasms/prevention and control"[Mesh] OR “Pancreatic Neoplasm” OR “Pancreas Neoplasm” OR “Pancreatic Cancer” OR “Pancreatic Adenocarcinoma” OR “Familial Pancreatic Cancer” OR “Familial Pancreatic Adenocarcinoma”) AND ("Early Detection of Cancer"[Mesh] OR “Pancreatic Cancer Surveillance” OR “Pancreatic Cancer Screening” OR “Pancreatic Screening” OR “Pancreas Surveillance” OR “Cancer Early Detection” OR “Cancer Screening” OR “Cancer Screening Test” OR “Early Diagnosis of Cancer” OR “Cancer Early Diagnosis” OR “Early Detection”) AND ("Cost-Effectiveness Analysis"[Mesh] OR “cost-effectiveness” OR “cost effectiveness” OR “economic analysis” OR “economic evaluation” OR “economic model” OR “cost analysis” OR “cost effectiveness” OR “cost-effectiveness analysis”)
